# Postprandial Metabolism and Appetite Do Not Differ between Lean Adults that Eat Breakfast or Morning Fast for 6 Weeks

**DOI:** 10.1093/jn/nxx004

**Published:** 2018-01-25

**Authors:** Enhad A Chowdhury, Judith D Richardson, Kostas Tsintzas, Dylan Thompson, James A Betts

**Affiliations:** 1 Department for Health, University of Bath, Bath, UK; 2 School of Life Sciences, Queen's Medical Centre, University of Nottingham, Nottingham, UK

**Keywords:** breakfast skipping, appetite hormones, insulin sensitivity, second-meal effect, energy intake

## Abstract

**Background:**

It remains unknown whether sustained daily feeding-fasting patterns modify the acute response to specific feedings on a given day.

**Objective:**

We conducted a randomized controlled trial to establish if daily breakfast consumption or fasting until noon modifies the acute metabolic and appetitive responses to a fixed breakfast and ad libitum lunch.

**Methods:**

With the use of a parallel group design, we randomly assigned 31 healthy, lean men and women (22–56 y) to 6 wk of either consuming ≥700 kcal of self-selected items before 1100 or fasting (0 kcal) until 1200 daily. Following 48 h of diet and physical activity standardization, we examined metabolic and appetite responses to a standardized breakfast and ad libitum lunch before and after the intervention. Data were analyzed using 3- and 2-way ANCOVA.

**Results:**

Systemic concentrations of energy balance regulatory hormones total and acylated ghrelin, leptin, and peptide tyrosine-tyrosine) responded similarly to breakfast and lunch before and after 6 wk of either morning fasting or regular breakfast, with the exception of a tendency for increased glucagon-like peptide-1 concentrations from baseline to follow-up in the Breakfast Group compared with a decrease over that period in the Fasting Group [*P* = 0.06, partial eta squared value (ƞ^2^) = 0.16]. Subjective appetite sensations also did not differ over the course of the day, and ad libitum energy intake at lunch was not systematically affected by either intervention, decreasing by 27 kcal (95% CI: −203, 149 kcal) with fasting and by 77 kcal (95% CI: −210, 56 kcal) with breakfast. Similarly, glycemic, insulinemic, lipemic, and thermogenic responses to breakfast and lunch were very stable at baseline and follow-up and, thus, did not differ between treatment groups.

**Conclusions:**

Our results indicate that a sustained period of either extended morning fasting or eating a daily breakfast has minimal effect upon acute metabolic and appetite responses in lean adults. This trial was registered at www.isrctn.org as ISRCTN31521726.

## Introduction

Infrequent or insufficient breakfast consumption is correlated with a greater risk of becoming overweight or obese (1–3). Cross-sectional evidence associating breakfast consumption and energy intake (EI) is equivocal, with some reports indicating no difference between breakfast consumers and skippers (3, 4) and others reporting increased EI among those that consume breakfast (5, 6). The majority of evidence from randomized controlled trials suggests that breakfast skipping leads to reduced EI (7) in both free-living (8–10) and laboratory settings (11–15).

Despite this evidence relating to breakfast skipping and EI, it remains unknown whether sustained exposure to daily morning fasting or feeding causes adaptations in appetite and metabolic responses. This is particularly pertinent because it has been suggested that self-reported breakfast-consumption habits can affect acute responses to morning fasting or feeding, potentially because of entrainment of metabolic and appetitive regulatory systems (16). However, much like other cross-sectional evidence linking breakfast consumption and positive health, it needs to be established if these results are causally attributable to breakfast habit per se or other healthful behaviors that cluster with breakfast consumption.

Previous interventions examining breakfast skipping (17) and morning fasting (8, 18) in lean individuals have examined health markers such as blood lipids and insulin sensitivity. Of these studies, 2 experiments found no effect of either a 1-wk intervention upon glucose and insulin (18) or a 6-wk intervention upon insulin sensitivity assessed by using the oral-glucose-tolerance test (OGTT) (8). However, another investigation assessed postprandial insulin sensitivity by using a mixed-macronutrient test drink (17), with the authors reporting reduced insulin sensitivity following a 2-wk breakfast-skipping intervention in lean women. Further, recent evidence from obese individuals suggests improved insulin sensitivity (which was assessed using the OGTT) as a result of 6-wk daily breakfast, as compared with those fasting until 1200 (19). Therefore, the effect of altered morning feeding frequency upon insulin sensitivity is currently unclear.

Beyond this outstanding question relating to the effect of morning feeding pattern upon insulin sensitivity, the aforementioned studies did not assess if appetite hormone responses to feeding were affected by the interventions. Evidence from studies characterizing appetite hormone responses to more chronic (8 wk) interventions based upon manipulating feeding frequency (but not the presence or absence of breakfast) (20, 21) and the macronutrient composition of diet (22) have reported no alteration of acute appetite hormone responses. Therefore, despite cross-sectional evidence that habitual morning feeding patterns may be linked to metabolic and appetitive responses, no randomized controlled trial has examined changes in acute appetite with altered breakfast consumption. By examining the effects of a randomly assigned and sustained morning-fasting or feeding intervention, it can be established if there are genuine effects of chronic breakfast consumption or breakfast skipping on acute metabolic regulation.

The present study therefore will investigate for the first time, to our knowledge, whether chronic exposure (6 wk) to a morning-fasting or daily breakfast regimen causes adaptations in ad libitum lunch intake, acute appetite regulation, and metabolic responses in lean men and women. We hypothesize that postprandial insulin sensitivity will be reduced in those undertaking morning fasting.

## Methods

### Participants

Thirty-one healthy, lean men (*n* = 12) and women (*n* = 19) aged 22–56 y took part in the study. Participants were recruited via local advertisement from South West England and were initially assessed for eligibility based upon a BMI (in kg/m^2^) of 18–25 and then later classified as lean based upon DXA-derived fat mass indexes of ≤7.5 kg/m^2^ (men) and ≤11 kg/m^2^ (women). In accordance with eligibility criteria previously set out (23), participants reported being weight stable (±1 kg within the past 6 mo) and adhering to a standard sleep-wake cycle (e.g., no shift workers) and not anticipating any change in lifestyle during the study period. Participants were free of metabolic disorders, with those participants that were premenopausal either menstruating regularly or following their chosen contraceptive method (i.e., pill or implant) for >6 mo. The sample size for this study was based on the estimates for the wider project described previously in our protocol paper (23). The program of work was estimated based on the number of individuals in each treatment group (∼14) required to confer a 90% probability to detect a 646-kcal increase in physical activity energy expenditure during the free-living component of the study, with the use of a 2-tailed *t*-test with an α level of 0.05. Thirty-five individuals completed the first acute breakfast trial before the free-living intervention. After this visit, 1 individual dropped out before the free-living intervention began; 1 individual was excluded from the Fasting Group because of noncompliance with the intervention protocol; and 1 individual from each intervention group failed to complete the follow-up acute breakfast trial after the intervention period. Within the study cohort, there was a mixture of regular breakfast consumers (classified by self-report as >50 kcal intake within 2 h of waking on ≥4 d of the week) and nonconsumers, with this factor included as a strata in the randomization plan. Characterization of habitual breakfast consumption is arbitrary, but this cut-off was designed to identify individuals who demonstrated a relatively extreme form of habitual breakfast skipping. The EI cut-off of 50 kcal was designed such that an individual could consume a hot drink such as tea or coffee with some milk, but any food intake would surpass the breakfast-consumption boundary. Characteristics of participants are presented in **Table 1**.

**TABLE 1:** Participant characteristics of lean men and women that undertook feeding trials before and after 6 wk of breakfast consumption or morning fasting^1^

Characteristic	Breakfast group	Fasting group
*n*	15	16
Age, y	36 ± 12	35 ± 10
Body mass, kg	66.6 ± 8.4	66.0 ± 7.9
BMI, kg/m^2^	21.6 ± 1.7	22.7 ± 2.3
Fat mass index, kg/m^2^		
All	5.1 ± 1.9	5.7 ± 2.3
Women	6.1 ± 1.8	6.7 ± 2.1
Men	3.6 ± 1.0	4.1 ± 1.6
Habitual breakfast consumers, *n*	11	14
Women, *n*	9	10

^1^Values are *n* or means ± SDs. Fat mass index is calculated as DXA-derived total fat mass divided by height squared.

Written informed consent was obtained from all participants, with the Ethical Approval for the study obtained from the NHS Bristol Research Ethics Committee in accordance with the ethical standards laid down in the 1964 Declaration of Helsinki and its later amendments. The study is registered with Current Controlled Trials [ISRCTN31521726].

### Study design

**FIGURE 1:**
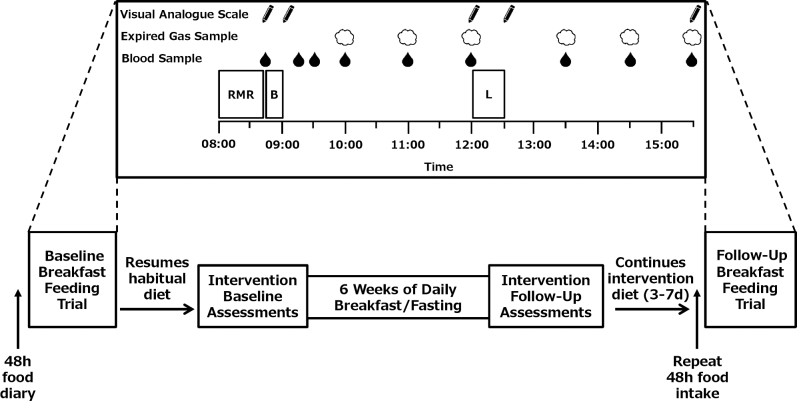
Schematic showing study design. In menstruating women, the 6-wk intervention commenced 2 wk after the baseline assessments so that the intervention follow-up visit was conducted in the follicular phase (3–10 d after onset of menses). In some cases, the first breakfast-feeding trial occurred after this baseline assessment but before the intervention beginning, such that it was also within the follicular phase. The 48-h food diary completed before the first breakfast feeding trial and repeated before the follow-up breakfast feeding trial conformed to the participants’ assigned intervention. The top half of the figure depicts the experimental protocol during breakfast feeding trial days. B, breakfast consumption; L, ad libitum lunch; RMR, resting metabolic rate.

The study was part of a wider program of research entitled the Bath Breakfast Project, involving 3 distinct phases in the same individuals (23), from which we have previously published the first 2 elements. These were a short cross-over laboratory experiment contrasting the acute metabolic responses to breakfast compared with fasting on a given day (14) that demonstrated incomplete energy compensation at lunch after extended morning fasting but greater insulin and glycemic response during the afternoon relative to breakfast consumption. Subsequently, a free-living component contrasting the chronic behavioral and metabolic responses during 6 wk of daily breakfast consumption with those of 6 wk of morning fasting was undertaken (8). This element resulted in little difference in health markers between the 2 groups, with lower reported EI but also physical activity energy expenditure in those fasting until 1200 each day. Here we report the culmination of this program of work by following-up the same population of lean adults and repeating their laboratory-based breakfast trial after completion of the 6-wk dietary intervention (**Figure 1**). In this way we can examine whether sustained adherence to a given morning feeding pattern can result in a chronic adaptation in the acute response to daily meals.

### Intervention

During the 6-wk intervention, participants were randomly assigned (1:1 allocation ratio) into 1 of 2 groups. One group was prescribed an EI of ≥700 kcal before 1100 daily, with at least half consumed within 2 h of waking (Breakfast Group). There were no stipulations on the composition of this breakfast, but to facilitate compliance with the intervention, individuals in this group were provided with information about the energy content of various breakfast items and had scales for weighing food items. The other experimental group extended their overnight fast by abstaining from ingestion of energy-providing nutrients (i.e., plain water only) until 1200 each day (Fasting Group). Beyond the morning period, all other lifestyle choices were allowed to vary naturally. In addition to the food diaries maintained during weeks 1 and 6 of the intervention, continuous glucose monitors were used to document glycemic responses to the intervention and verify compliance with the assigned interventions. Sufficient length for the intervention was determined as 6 wk, as alterations in insulin sensitivity with feeding frequency and timing interventions have been observed in as little as 2 wk (17, 24), with differences in weight change apparent in as little as 4–6 wk (25, 26). The effect of this intervention period upon components of free-living energy balance and other health measures has been reported previously (8). The randomization of participants was stratified according to baseline breakfast habits to control for the distribution of habitual breakfast consumers and nonconsumers in each group. The experimenters conducting analyses were unblinded to group assignment because many measures required direct interaction with unblinded participants or the group allocation was immediately evident in the data (e.g., diet records), and these same experimenters conducted tissue and data analyses.

### Standardization of participants before the second feeding trial

Between the 2 laboratory visits for feeding trials, participants undertook their assigned dietary intervention for 6 wk with pre- and postintervention visits to assess body composition changes and glycemic control using the OGTT (8). After this postintervention visit, within 3–7 d, participants returned to the laboratory for their second breakfast-feeding trial. During the intervening period, participants continued to follow their assigned intervention dietary regimens (i.e., ≥700 kcal EI by 1100 or 0 kcal until 1200 daily), such that any chronic adaptations to their feeding regime were not affected by a change of dietary practices before their follow-up breakfast-feeding trial. Therefore, the total adherence to the participants’ assigned intervention diet was 6–7 wk. In addition, participants also completed 48 h of diet standardization that corresponded to their assigned free-living intervention, and refrained from strenuous physical activity before both of the feeding trials.

### Protocol for laboratory visits

The experimental protocol is identical to the breakfast feeding trials outlined in previous studies from this program of work (13, 14) and analytical procedures as described in full in our protocol paper (23). In brief, participants reported to the laboratory at 0800 ± 1 h, upon which adherence to standardization measures were confirmed verbally. Participants then voided and had body mass measured in light clothing (Seca 873; Vogel and Halke). On their first visit to the laboratory, before commencement of their intervention, resting metabolic rate (RMR) was assessed in a supine position by repeated 5 min expired gas samples over ∼30 min, according to best practice (27). After their intervention on the second feeding trial, this procedure was repeated. A cannula was then inserted into an antecubital vein, with a baseline sample of 15 mL obtained and further samples acquired at regular intervals throughout the day. Participants were then provided with a breakfast. Blood samples were then taken at 15 min, 30 min, and 1 h after completion of the breakfast period. One hour after the breakfast period had ended, an expired gas sample was also obtained for assessment of diet-induced thermogenesis and substrate oxidation. Blood and gas samples were then obtained hourly until 3 h after breakfast, at which point an ad libitum lunch was provided. Upon completion of the lunch period (30 min), samples of blood and expired gas were obtained each hour for a further 3 h. Participants also completed visual analogue scales relating to appetite at selected time points throughout the day. During the day, participants remained sedentary and completed quiet activities such as reading, watching television, and typing.

### Breakfast

The breakfast consisted of Corn Flakes (Kellogg's), 2% fat milk (Sainsbury's), toasted white bread (Braces), margarine (Unilever “I Can't Believe It's Not Butter”), and fresh orange juice (Sainsbury's). For provision of additional sugar, participants were given the choice of either white sugar added to cornflakes or seedless raspberry jam (Sainsbury's) on their toast, or an isocaloric combination of both. The overall percentages of energy from macronutrients in the breakfast were 69.6% carbohydrate, 17.5% fat, and 12.9% protein. The breakfast was provided in quantities that contained 0.06 g carbohydrate/kcal of each individual participant's measured daily RMR, resulting in EI of 472 ± 67 kcal and 460 ± 47 kcal in the Breakfast and Fasting Groups, respectively. Participants were first provided with cereal, and then at 5-min intervals, toast, and, finally, orange juice, with all of the breakfast consumed within 15 min to standardize any effects of eating rate upon appetite hormones (28).

### Ad libitum lunch

The ad libitum lunch test meal consisted of cooked (i.e., wet weight) penne pasta (Sainsbury's) and tomato sauce (Ragu); prepared at a ratio of 1:1 uncooked mass. The overall percentages of energy from macronutrients for the lunch were 79% carbohydrate, 14% fat, and 7% protein. During the first trial, participants were allowed ad libitum intake of plain water during lunch; this volume was subsequently replicated on their second visit. Participants were left alone during the lunch, with a recorded message played before beginning consumption, asking them to eat until they had satisfied their hunger. Pasta was provided in a large bowl containing 1 kg of cooked pasta, which was replenished every 10 min during the lunch period to minimize visual feedback and prevent any tendency to finish the portion provided. The mass of pasta consumed during the lunch was recorded, and EI was calculated using the manufacturer's nutritional information.

#### Expired gas analysis

Douglas bags were employed to obtain expired air samples with samples for RMR collected in line with guidance for best practice (27, 29). Rates of both oxygen utilization (VO_2_) and carbon dioxide production (VCO_2_) were used to calculate energy expenditure (30) corrected for urinary nitrogen excretion (31):

(1) \begin{eqnarray*} {\rm{Energy}}\,{\rm{Expenditure}} &=& \left( {3.941 \times {\rm{V}}{{\rm{O}}_2}} \right) + \left( {1.106 \times {\rm{VC}}{{\rm{O}}_2}} \right) \nonumber\\ && +\, \left( {2.17 \times {\rm{Nitrogen}}\,{\rm{Excretion}}} \right) \end{eqnarray*}

#### Blood sampling and analysis

Blood was sampled via intravenous cannula inserted into the veins of the antecubital region of the arm. Blood was collected and stored as serum or plasma by using standard methods, apart from samples for analysis of acylated ghrelin, for which blood was treated with proteases to prevent degradation as described previously (14). Total ghrelin (intra-assay CV, 4.0%; inter-assay CV, 7.8%), acylated ghrelin (intra-assay CV, 4.2%; inter-assay CV, 11.3%; Bertin Pharma, Montigny le Bretonneux, France), and peptide tyrosine-tyrosine (PYY) (intra-assay CV, 4.3%; inter-assay CV, 11.1%) assays were conducted using plasma. Leptin (intra-assay CV, 3.4%; inter-assay CV, 6.4%) (R&D Systems Inc., Abingdon, UK) and insulin (intra-assay CV, 4.7%; inter-assay CV, 12.5%) (Mercodia AB, Uppsala, Sweden) assays were conducted using serum. Assays employed were commercially available ELISA conducted following manufacturer instructions, with all samples batch analyzed upon study completion and samples from each participant assayed on the same plate. Plasma samples were analyzed for nonesterified fatty acids (NEFA) (intra-assay CV, <5%; inter-assay CV, <5%), glucose (intra-assay CV, <5%; inter-assay CV, <6%) and urea (intra-assay CV, <5%; inter-assay CV, <3%), using a Daytona automated analyzer (Randox Laboratories, Crumlin, NI) according to manufacturer guidelines using commercially available immunoassays (Randox Laboratories, Crumlin, NI).

#### Urine collection

Urine was collected in containers with 5 mL of 10% thymol isopropanol used as a preservative. The urine collected during a measurement period was thoroughly mixed, and a 1-mL aliquot was abstracted and stored at −80°C. Urinary urea concentrations for use in calculations to estimate urinary nitrogen excretion (and, therefore, non-protein RER) were obtained using a commercially available immunoassay as described above for plasma.

#### Appetite sensations

Paper visual analogue scales of 100 mm length were employed to assess subjective appetite. These scales were completed before and after breakfast, before and after lunch, and following a 3-h postprandial period after lunch. Participants marked a response to questions assessing desire to eat, hunger, fullness, and prospective consumption with anchor phrases on the ends of each of the scales (e.g., “not at all hungry” compared with “as hungry as I have ever felt”). Higher scores are indicative of greater sensations. A composite appetite score (32) was also calculated using the following formula: (Desire to Eat + Hunger + (100-Fullness) + Prospective Consumption) / 4 to give an overall indication of appetite at the measured time points.

### Statistical analysis

For comparison of time-series variables measured over the course of the day (e.g., appetite hormones), 3-way mixed model ANCOVA using AUC of the variable of interest from the baseline trial as a covariate were employed to identify interactions between group (i.e., breakfast compared with fasting), trial (i.e., baseline compared with follow-up), and time-point (i.e., time of day), all independent of deviations from a normal distribution (33) but with Greenhouse-Geisser corrections applied to intra-individual contrasts for ɛ < 0.75, and the Huynh-Feldt correction applied for less severe asphericity (34). Where relevant, to provide a fuller appreciation of any potential changes in response to the interventions, time courses were also expressed as peak, nadir and fasting concentrations specific to each participant (e.g., the average of the peak concentrations within each participant, irrespective of when this occurred), with these summary statistics subsequently analyzed by 2-way ANCOVA using AUC of the variable of interest from the baseline trial as a covariate. Partial eta squared values (ƞ^2^) are displayed to illustrate effect size for statistically significant and near significant interactions. Data are presented in the text as mean ± SD; figures display mean with error bars representing SEM. All statistical analyses were conducted using IBM SPSS statistics version 22 (IBM, New York).

## Results

### Mass and RMR

From their preintervention to postintervention breakfast trial, the body mass of both groups was stable within 0.35 kg. RMRs before the intervention were similar between the Fasting Group and the Breakfast Group (1402 ± 149 compared with 1452 ± 201 kcal/d) and were not differently affected by the intervention, such that the values after intervention for the Fasting Group (1417 ± 129 kcal/d) and the Breakfast Group (1421 ± 187 kcal/d) were stable within 35 kcal/d from before to after the intervention.

### Energy intake

**FIGURE 2:**
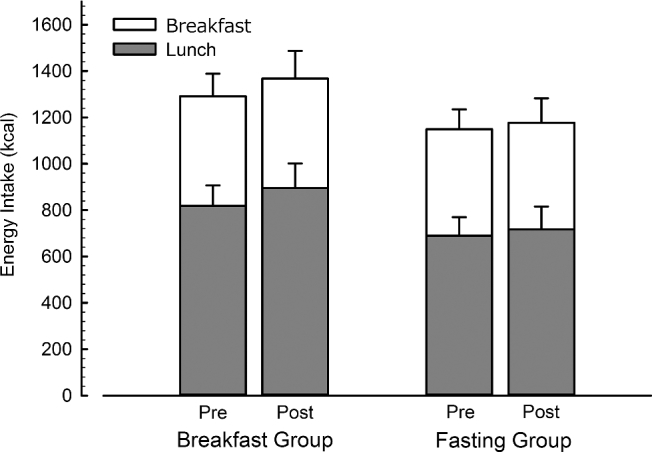
Energy intake in lean men and women during the feeding trial before and after 6 wk of daily breakfast or fasting. Values are means, with the error bars on the gray portion of the stack representing the SEMs of the energy intake at lunch, and the error bars on the white portion the SEMs of the energy intake of the whole day (*n* = 31). Post, after 6 wk of daily breakfast or fasting; Pre, before 6 wk of daily breakfast or fasting.

Breakfast intake was prescribed, with the breakfast provided for both groups based on RMR similar (Breakfast Group, 472 ± 67 kcal compared with the Fasting Group, 460 ± 47 kcal; *P* = 0.56) and did not change from pre- to postintervention levels. EI at lunch was not different between groups or trials, and there was no evidence of a trial x group interaction (all *P* > 0.2). Intake including the prescribed breakfast was 1150 ± 341 kcal before the intervention and 1177 ± 422 kcal after the intervention in the Fasting Group. In the Breakfast Group, intake was 1291 ± 377 kcal before the intervention and 1368 ± 461 kcal after the intervention (**Figure 2**).

### Systemic metabolites

**FIGURE 3:**
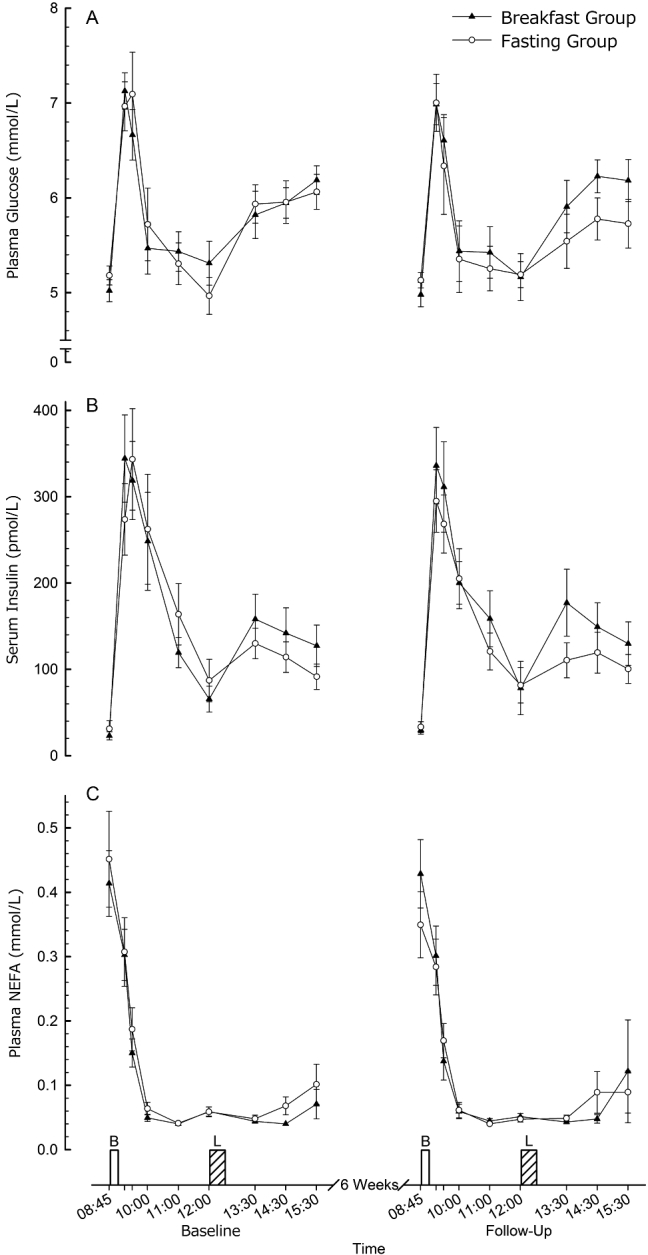
Plasma glucose (A), serum insulin (B), and plasma NEFA (C) responses to feeding in lean men and women before and after a 6-wk intervention in the Breakfast and Fasting Groups. Values are means ± SEMs (*n* = 31). B, prescribed breakfast; L, ad libitum lunch; NEFA, nonesterified fatty acid.

Blood plasma concentrations of glucose, NEFA, and serum insulin all responded as would be expected to feeding at breakfast and lunch, both at baseline and follow-up (**Figure 3****A, B, and C**, respectively). Analysis of covariance revealed no interaction effects such that the magnitude of this response over time did not differ between the Breakfast and Fasting Groups. Specific comparisons in relation to individual peak, nadir, or fasted concentrations also did not reveal any differences between the Breakfast and Fasting Groups at any time-point.

### Energy balance regulatory hormones

**FIGURE 4:**
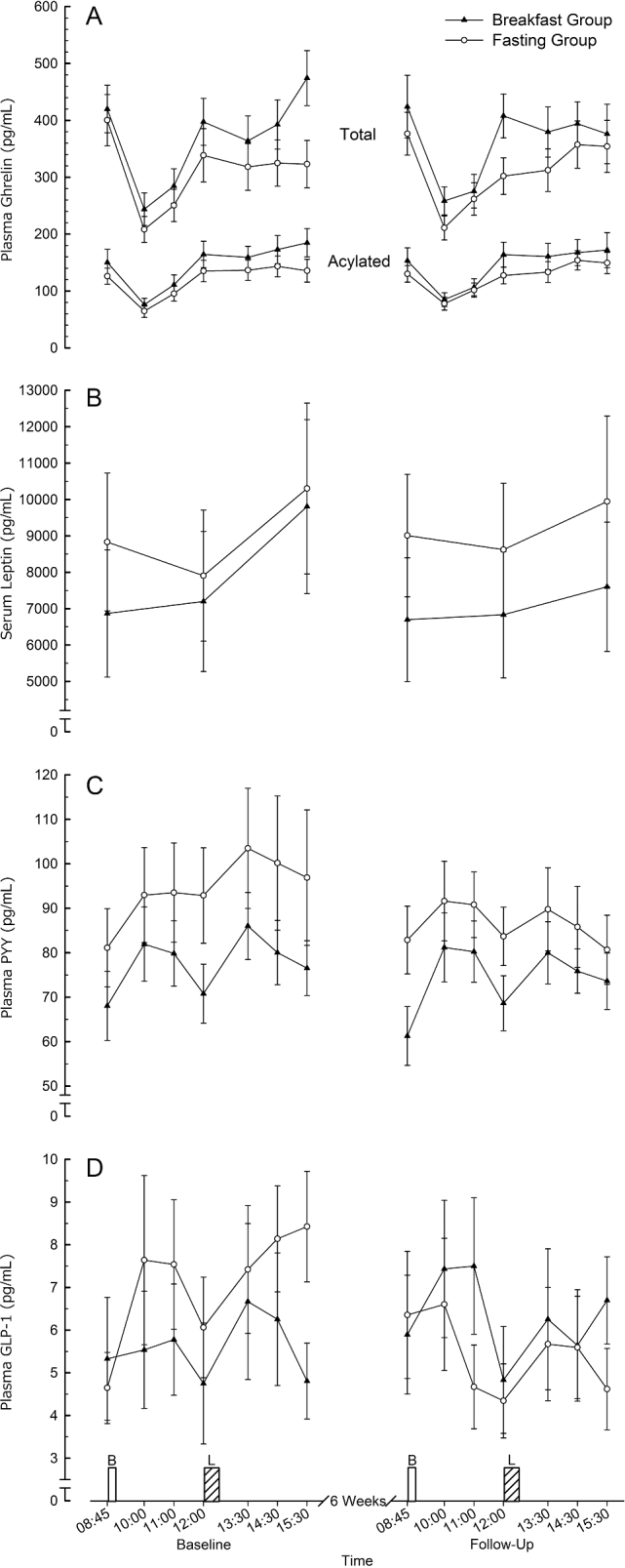
Plasma total and acylated ghrelin (A), serum leptin (B), plasma PYY (C) and plasma GLP-1 (D) responses to feeding in lean men and women before and after a 6-wk intervention in the Breakfast and Fasting Groups. Values are means ± SEMs (*n* = 31). B, prescribed breakfast; GLP-1, glucagon-like peptide 1; L, ad libitum lunch; PYY, peptide tyrosine-tyrosine.

**Figure 4** illustrates the time-course of response for a number of hormones involved in the regulation of appetite and energy balance. All acutely responded as would be expected to the feeding stimuli in each trial, but without any difference in the magnitude or time-course of these responses between treatment groups from baseline to follow-up for any parameter except for GLP-1, which exhibited a tendency for a trial x group interaction effect (*P* = 0.06, ƞ^2^ = 0.16; Figure 4D). This is consistent with a similar observation for individual peak GLP-1 concentrations (*P* = 0.05, ƞ^2^ = 0.14). Specific contrasts of fasted, peak, or nadir values did not reveal any interaction effects for any other parameters. Plasma adiponectin exhibited high variability in measurement, with a tendency for a trial × group interaction (*P* = 0.07, ƞ^2^ = 0.12). The mean concentration cross baseline and follow-up trials was 9472 ± 3343 ng/mL and 9582 ± 3284 ng/mL in the Breakfast Group and 7919 ± 3426 ng/mL and 9097 ± 4082 ng/mL in the Fasting Group, respectively.

### Appetite sensations

**FIGURE 5:**
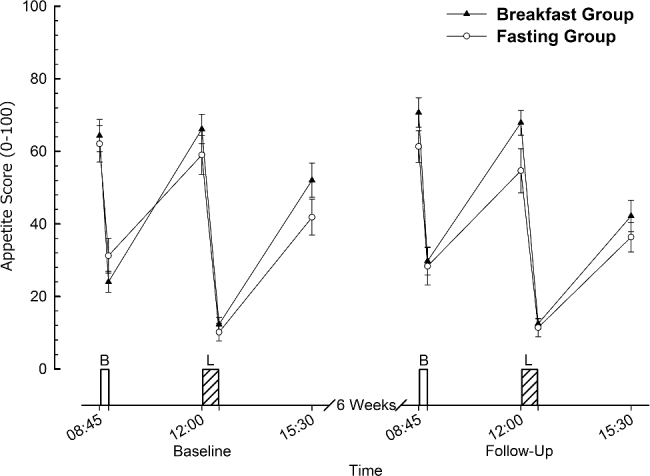
Appetite score during feeding trials in lean men and women before and after a 6-wk intervention in the Breakfast and Fasting Groups. Values are means ± SEMs (*n* = 31). B, prescribed breakfast; L, ad libitum lunch.

The composite appetite score calculated from visual analogue scales indicated that appetite varied over time (*P* < 0.01), but there were no other main effects or interactions (**Figure 5**; *P* > 0.1). Fasting appetite scores were not different between groups or trials, and no interaction effects were apparent (all *P* > 0.2). For the subcomponents of the appetite score (desire to eat, hunger, fullness, and prospective consumption), there were main effects of time (all *P* < 0.01), but no main effects of trial or group (all *P* > 0.1) for any component. Similarly, there were no interaction effects for any of the ratings apart from a trial x group interaction for fullness (*P* = 0.04). There were also no main effects or interactions for fasted ratings (all *P* > 0.1).

### Diet-induced thermogenesis (DIT)

**FIGURE 6:**
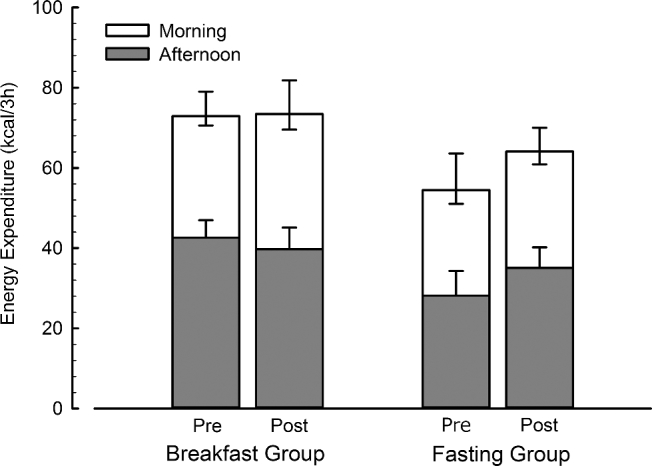
Energy expenditure above rest during feeding trials in lean men and women before and after 6 wk of daily breakfast or fasting. Values are the means, and error bars reflect the SEMs. Asymmetric error bars are plotted on the morning section of each stack. The negative portion of these error bars reflects the SEM of the morning period, and the positive portion the SEM of the whole day (*n* = 31). Post, after 6 wk of daily breakfast or fasting; Pre, before 6 wk of daily breakfast or fasting.

The post-prandial increase in resting metabolism measured from gaseous exchange was consistent between baseline and follow-up and between interventions (**Figure 6**). Accordingly, there were no main effects of trial or group nor any interaction of trial x group for the thermogenic response either to breakfast (all effects *P* ≥ 0.2), the ad libitum lunch (all effects *P* > 0.1), or when considered over the entire trial period (all effects *P* > 0.1).

## Discussion

The present study is the first to examine acute EI and metabolic and appetite responses in a laboratory-based protocol following a sustained intervention of morning fasting or daily breakfast consumption in lean adults. Contrary to our hypothesis, glycemia and insulinemia following feeding were not increased by the prolonged fasting intervention. Following a standardized breakfast, there was no adaptation of EI at an ad libitum lunch, and most appetite-hormone responses across the day were similarly unaffected because of either daily fasting or breakfast consumption. The present study indicates that sustained morning fasting does not stimulate chronic adaptations that increase hunger or EI or elicit negative metabolic consequences to standardized breakfast and ad libitum lunch.

It has previously been reported that a 2-wk period of daily breakfast eating in lean women reduced the insulin response to a mixed-macronutrient drink, whereas delaying the first feeding of the day until 1100 resulted in a significantly greater insulin response to the test drink (17). Here, we find no evidence of either a significant reduction with breakfast consumption or detrimental increases in insulin response to meal consumption after 6 wk of morning fasting. This is particularly interesting because the changes observed in the aforementioned work were induced in only 2 wk of intervention, compared with 6 wk in the present study. We have previously reported that the response to glucose ingestion was unaffected in lean individuals undertaking the more prolonged interventions employed here (8), but, in contrast, the insulinemic response in obese individuals who had been consuming breakfast daily was reduced, whereas among those who had been fasting each morning it was increased (19). In combination, these results suggest that the effect upon insulin sensitivity of differing chronic morning feeding patterns are variable and may be affected by the specific nature of the prescribed intervention, measurement context (e.g., meal compared with glucose ingestion), and characteristics of study population (e.g., lean or obese).

The current investigation also examined a second self-regulated feeding occasion. Although these lunches were of identical composition, they were not of the same size before that they were after intervention (as the lunch meals were ad libitum to identify any alterations in food consumption), and the magnitude of difference in both groups was an increase of <80 kcal, which allows some comparison of insulin response to reasonably equivalent meals. After this second meal, the insulin response during the afternoon was stable from baseline to follow-up in both groups. This confirms that postprandial insulin responses are not increased in lean adults who followed a daily morning-fasting intervention whether at the first meal of the day or having already eaten. As acutely elevated NEFA concentrations can cause reduced insulin sensitivity owing to impaired insulin signaling (35), it is relevant to note that NEFA concentrations throughout the testing day were also unaffected after the fasting intervention.

To the best of the authors’ knowledge, no study to date has investigated the effect of a sustained morning-fasting intervention on the metabolic and hormonal factors that regulate and respond to acute feeding in lean adults (i.e., whether daily exposure to morning fasting causes adaptation of appetite responses examined within a day). Some authors have reported benefits for acute appetite regulation with breakfast consumption (36, 37), but our findings from this longer-term intervention are not consistent with these reports, as exposure to a morning-fasting intervention did not lead to increased intake at the ad libitum lunch in the present study. It has been observed that the nature of an ad libitum meal promotes overconsumption (38), and, therefore, it could be that the test meal we employed in our study may not have been sensitive to alterations in hunger. However, the positive results mentioned above from other authors have utilized a similar ad libitum meal design. Additionally, in our study, appetite ratings before lunch were unchanged after 6 wk of daily fasting in this study, and the response of the acute hunger hormone acylated ghrelin were virtually identical at follow-up, further substantiating that our test meal results may accurately represent the appetite in our participants. Therefore, neither daily self-selected breakfast nor, more surprisingly (as it was unaccustomed in more participants), morning fasting over a 6-wk period induces any adaptations in lunch EI or subjective appetite in lean individuals.

The absence of altered lunchtime EI to either intervention is also consistent with the stability of appetite hormone responses before and after the interventions. Few studies have examined acute appetite hormone responses after a chronic dietary intervention. These investigations have examined the effects of interventions manipulating feeding frequency (20, 21) and macronutrient composition of diet (22) upon acute appetite hormone responses to feeding. In concordance with the current work, none of these prior investigations has reported alterations in acute appetite hormone responses from pre- to postchronic intervention. Although it has been reported that exercise- (39) and diet-induced (40) weight-loss interventions can modify postprandial appetite hormone concentrations, it appears that these hormonal responses are relatively resistant to change in the context of altered feeding patterns without substantial weight change.

One appetite hormone that has been suggested to be modifiable through altered feeding patterns is the orexigenic hormone ghrelin. It has been shown that ghrelin concentrations are entrained to habitual feeding patterns, with peaks synchronized to the expected time of feeding (41). Other authors have reported that shifting breakfast intake from 0700 to 0900 for 2 wk can delay the peak in ghrelin concentration (42). Following this line of reasoning, it might be expected that ghrelin would peak later in the morning or be reduced at the start of the day after a fasting intervention, but this was not the case in the current intervention. This may well be due to the duration of the overnight fast resulting in a “maximal” drive to eat independent of habitual patterns and, potentially, owing to expectancy of feeding in the laboratory environment.

Some considerations should be accounted for when interpreting the present results. First, the participants assigned to breakfast in this study consumed a self-selected breakfast that was defined by energy content because we were primarily interested in the presence or absence of food intake per se. Accordingly, we cannot make any comments relating to effects of specific compositions of breakfast, and it is perfectly plausible that certain compositions such as protein-rich (37), low GI (43), or high-fiber (44) breakfast interventions might have different effects. Along similar lines, the meals used during the test day were relatively carbohydrate-rich, so different meal compositions should also be examined. Second, while we can conclude that our intervention did not modify the response to repeated meals, we did not conduct a follow-up morning-fasting trial and, therefore, cannot be sure that the feeding or fasting interventions did not modify the response to morning fasting. Finally, our results should not be applied to other groups, such as obese individuals, as differences in acute appetite regulation may be apparent (45, 46). In addition, beneficial effects of a high-protein breakfast intervention have been demonstrated in type 2 diabetics (47).

An important outstanding question regarding the role of breakfast in promoting energy balance and health has been whether longer-term breakfast habits can adjust how we respond to a breakfast on a given day (i.e., a chronic adaptation in the acute response). This study provides no evidence that even the relatively extreme practice of remaining completely fasted until midday every day for 6 wk can modify the metabolic and appetitive response to repeated meals in healthy lean adults, indicating relative stability of these responses in the absence of weight change. Further to this, the present work suggests that study designs examining the acute responses to breakfast or fasting likely reflect accurate responses to these challenges and there is no need to “habituate” to a certain feeding pattern to modify acute metabolic responses. Finally, this study suggests that prolonged adherence to extended daily morning fasting does not cause detrimental effects upon acute metabolic regulation in lean adults.
